# Fc-Linked IgG *N*-Glycosylation in FcγR Knock-Out Mice

**DOI:** 10.3389/fcell.2020.00067

**Published:** 2020-03-03

**Authors:** Olga O. Zaytseva, Michaela Seeling, Jasminka Krištić, Gordan Lauc, Marija Pezer, Falk Nimmerjahn

**Affiliations:** ^1^Glycoscience Research Laboratory, Genos Ltd., Zagreb, Croatia; ^2^Division of Genetics, Department of Biology, University of Erlangen-Nürnberg, Erlangen, Germany; ^3^Faculty of Pharmacy and Biochemistry, Department of Biochemistry and Molecular Biology, University of Zagreb, Zagreb, Croatia

**Keywords:** Fcγ receptor, IgG *N*-glycan profile, immunoglobulin G, *N*-glycosylation, liquid chromatography–electrospray ionization–mass spectrometry

## Abstract

Immunoglobulin G (IgG) is the most abundant immunoglobulin isotype in the blood and is involved in the pathogenesis and progression of various diseases. Glycosylation of the IgG fragment crystallizable (Fc) region is shown to vary in different physiological and pathological states. Fc *N*-glycan composition can alter the effector functions of IgG by modulating its affinity for ligands, such as Fcγ receptors (FcγRs). However, it is not known whether IgG glycosylation is affected by the available repertoire of FcγRs, and if the Fc-linked *N*-glycome can compensate for modulation of the IgG–FcγR interaction. To explore this, we examined the subclass-specific Fc IgG glycoprofiles of healthy male and female FcγR knock-out mice on C57BL/6 and BALB/c backgrounds. We observed slight changes in IgG Fc *N*-glycan profiles in different knock-outs; however, it seems that the strain background and sex have a stronger effect on *N*-glycosylation of IgG Fc regions than the FcγR repertoire.

## Introduction

**FIGURE 1:**
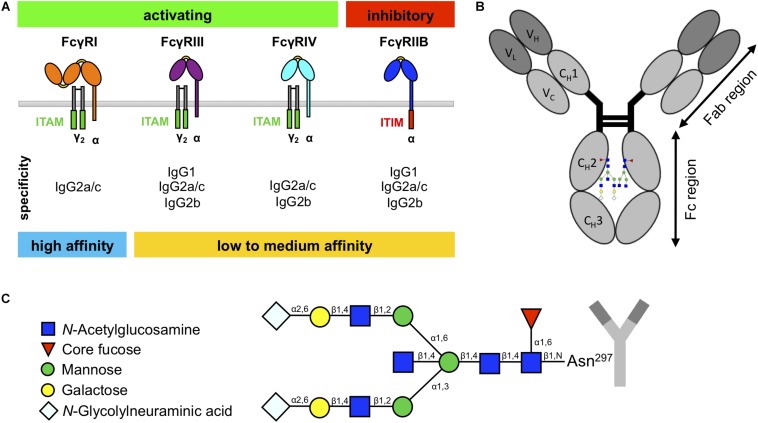
Murine fragment crystallizable (Fc)γ receptors (FcγRs) and immunoglobulin G (IgG) glycosylation. **(A)** Schematic overview of murine FcγRs with their specificity to IgG subclasses. Whereas the high-affinity FcγRI can bind monomeric IgG, the low- to medium-affinity FcγRs can only bind IgG in the form of immune complexes. **(B)** IgG molecules consist of an Fab region, which is important for antigen binding, and an Fc region, which is important for interaction with the FcγRs. **(C)** IgG has one glycan chain attached to the asparagine 297 (Asn^297^) on each constant heavy domain (C_*H*_2) in the Fc region. The sugar chain consists of a constant heptameric core structure containing mannose and *N*-acetylglucosamine residues. The core can be extended with core fucose and bisecting *N*-acetylglucosamine, and the branching arms (α6 and α3) can display a variable glycosylation pattern, containing galactoses and *N*-glycolylneuraminic acids.

Immunoglobulin G is the most abundant Ig isotype in the blood and plays a key role in defending the host against microbial infections but also in pathological events (such as inflammation) underlying different diseases. IgG can activate a variety of effector mechanisms, such as complement-dependent cytotoxicity, antibody-dependent cellular cytotoxicity, and phagocytosis (Klein-Schneegans et al., 1989; Woof and Burton, 2004; Schroeder et al., 2010). Many IgG effector functions are initiated through the binding of the IgG Fc domain of antibodies or immune complexes to specialized surface receptors, FcγRs, which are widely expressed on most hematopoietic cells (Unkeless et al., 1988; Nimmerjahn and Ravetch, 2008). In mice, these include three activating (FcγRI, FcγRIII, FcγRIV) and one inhibitory FcγR (FcγRIIB) (Figure 1A; Unkeless et al., 1988; Nimmerjahn and Ravetch, 2008). The majority of cells co-express the activating FcγRs and the inhibitory FcγRIIB on their surface, which allows setting a threshold for cell activation through differential FcγR signaling (Clynes et al., 1999; Nimmerjahn and Ravetch, 2010; Baerenwaldt et al., 2011; Nimmerjahn et al., 2015).

The murine IgG family consists of four different subclasses (IgG1, IgG2a/c, IgG2b, and IgG3), which show differential binding to FcγRs (Figure 1A; Nimmerjahn and Ravetch, 2005; Nimmerjahn et al., 2005). In contrast to IgG1, IgG2a/c, and IgG2b, IgG3 interact only very weakly with known FcγRs but is the most effective isotype to activate complement (Nimmerjahn and Ravetch, 2005; Bruhns, 2012; Sörman et al., 2014). Interestingly, there are known differences between mouse strains in the protein sequences of IgG1 and IgG2a/c heavy chains that occur in the proximity of the Fc *N*-glycosylation site (Zhang et al., 2012; Maresch and Altmann, 2016; de Haan et al., 2017; Zaytseva et al., 2018), and it has been hypothesized that the amino acid sequence of the heavy chain can impact the Fc glycosylation profile (Lund et al., 1996; Krištić et al., 2018).

Whereas most immunoglobulin isotypes show more than one glycosylation site within the Fc region, IgG only exhibits one glycan attached to the asparagine 297 of each C_*H*_2 domain (Figure 1B; Arnold et al., 2007; Böhm et al., 2012; Seeling et al., 2017). This biantennary glycan consists of a constant heptameric core structure containing three mannose residues and four GlcNAc residues and may contain additional core fucose and bisecting GlcNAc (Figure 1C). Additionally, the branching arms (α6 and α3) can display a variable glycosylation pattern consisting of terminal galactose and sialic acids (in mice *N-*glycolylneuraminic acid) (Arnold et al., 2007; Böhm et al., 2012; Seeling et al., 2017). Together with the fact that the two glycan moieties attached to each Fc domain can vary due to asymmetric glycosylation, this results in several hundred possible different IgG Fc glycosylation variants (Shields et al., 2002; Shinkawa et al., 2003; Arnold et al., 2007; Kao et al., 2015; Quast et al., 2015). In addition, recent studies in mice and humans suggest that different IgG subclasses show subclass-specific glycosylation patterns (Kao et al., 2015; de Haan et al., 2017; Zaytseva et al., 2018). More importantly, for some of these IgG glycosylation variants, an altered IgG activity was noted due to a modulation of binding to FcγRs (Nimmerjahn and Ravetch, 2005; Kao et al., 2015). The most notable example for this phenomenon is increased affinity of afucosylated IgG preparations for mouse FcγRIV and human FcγRIIIA (Nimmerjahn et al., 2015). In addition, IgG glycovariants rich in terminal sialic acid residues show a higher anti-inflammatory activity (Kaneko et al., 2006; Schwab et al., 2014; Ohmi et al., 2016). In line with these results, serum IgG and autoantibody glycosylation patterns are altered in autoimmune diseases and cancer in mice and humans and sometimes also associate with symptom severity, disease progression, and therapy response (Gudelj et al., 2018).

In this study, we investigated if a feedback regulation of the available repertoire of FcγRs and IgG glycosylation exists *in vivo*. We studied serum IgG glycosylation in 10-week-old male and female mice deficient for a single FcγR (FcγRI^–/–^, FcγRIIB^–/–^, FcγRIII^–/–^, FcγRIV^–/–^), all activating FcγRs (FcRγ^–/–^), or all FcγRs (FcRγ^–/–^FcγRIIB^–/–^) on the C57BL/6 background and for FcγRIIB^–/–^, FcRγ^–/–^, and FcRγ^–/–^FcγRIIB^–/–^ mice additionally on BALB/c background. IgG isolated from serum samples was digested with trypsin, and the resulting Fc-glycopeptides were analyzed by LC–ESI–MS. While we observed slight changes in IgG Fc *N*-glycan profiles in different knock-outs, our study shows that the genetic background and sex of the animals have a more pronounced effect on IgG Fc *N*-glycosylation than the FcγR repertoire.

## Materials and Methods

### Mice

Ten-week-old male and female mice deficient for a single FcγR (FcγRI^–/–^, FcγRIIB^–/–^, FcγRIII^–/–^, FcγRIV^–/–^), all activating FcγRs (FcRγ^–/–^), or all FcγRs (FcRγ^–/–^FcγRIIB^–/–^) on C57BL/6 background and FcγRIIB^–/–^, FcRγ^–/–^, and FcRγ^–/–^FcγRIIB^–/–^ mice on BALB/c background were used in this study. C57BL/6 and BALB/c mice were obtained from Elevage Janvier (Le Genest-Saint-Isle, France). Jeffrey Ravetch (Rockefeller University, NY, United States) provided all FcγR-deficient strains. All strains were bred in-house for several generations. Animals were maintained under specific pathogen-free conditions. The study was approved by the Ethical Committee of the District Government of Lower Franconia.

### Serum Samples

Blood was collected by retro-orbital bleeding through a sodium heparin-coated micro hematocrit capillary (Hirschmann Laborgeräte) into a *micro tube 1.1ml Z-Gel* (Sarstedt). After standing at room temperature for 30 min, the blood was centrifuged at 10 000 × *g* for 5 min, and serum was transferred to new tubes and stored at −20°C until analysis.

### ELISA

For quantification of serum IgG, the Bethyl Murine IgG ELISA Quantitation Kit (Biomol) was used according to the manufacturer’s instructions. Separate ELISAs were used to obtain concentrations for IgG2a, IgG2b, and IgG2c. To better compare data from ELISA with the glycosylation analysis, where this distinction was not possible, results were figured up to one value and are shown as “IgG2.” Optical density was measured with a VersaMax tunable microplate reader (Molecular Devices) at 450 and 650 nm.

### Analysis of IgG Fc-Linked *N*-Glycosylation With Liquid Chromatography–Electrospray Ionization–Mass Spectrometry

Immunoglobulin G was isolated from 500 μl of mouse serum on 96-well Protein G monolithic plates (BIA Separations) as described previously (Pezer et al., 2016). Briefly, serum samples were diluted in 1.4 ml of 1× PBS (prepared in-house from analysis-grade reagents), filtered through AcroPrep Advance 0.45 μm hydrophilic polypropylene (GHP) filter plates (Pall Corporation), bound to the protein G monolith in 1 × PBS, eluted in 0.1 M formic acid (Merck), and neutralized with 1 M ammonium bicarbonate (Merck). The sample layout on the plates was determined by block randomization to distribute samples from female and male animals of different strains evenly across the two plates. Each plate design included one negative control, standards, and duplicates to ensure quality control.

Isolated IgG was dried in the vacuum concentrator and dissolved in ultrapure water to achieve a concentration of approximately 0.3–0.8 μg/μl. Approximately 20 ng of the isolated IgG was pipetted into 0.2-ml skirted 96-well robotic plates (Thermo Fischer Scientific), digested with sequencing grade trypsin (Worthington), cleaned with RP SPE on Chromabond C18 ec beads (Marcherey-Nagel) and analyzed on a nanoACQUITY UPLC system (Waters) coupled to a Compact mass spectrometer (Bruker Daltonics) as described previously (Pezer et al., 2016). Tryptic glycopeptides corresponding to the Fc region of IgG were separated by LC in the gradient of 80% ACN (LC–MS purity, JT Baker, ThermoFischer Scientific) in 0.1% TFA (HPLC purity, Sigma-Aldrich) on a Halo C18 nano-LC column (150 mm × 75 μm i.d., 2.7 μm HALO fused core particles, Advanced Materials Technology), and mass spectra were recorded in positive mode. Separation between tryptic glycopeptides corresponding to IgG2a (in BALB/c mice)/IgG2c (in C57BL/6 mice) and IgG2b subclasses was not achieved with this method, and the respective *N*-glycoforms were quantified together. MS data were extracted using LaCyTools v1.0.1 b.7 software, glycopeptide compositions were assigned based on *m*/*z* values (Supplementary Table S1) and isotopic distributions for doubly and triply protonated ions (Jansen et al., 2016). Peaks with S/N ratio <9, isotopic peak quality >40%, or *m*/*z* values after calibration noticeably deviating from the theoretical ones were not integrated.

### Statistical Analysis and Representation of the Data

#### ELISA

Due to the group size, all ELISA data were considered not normally distributed. Thus, either Mann–Whitney test for two groups or Kruskal–Wallis test followed by *post hoc* Dunn test (multiple comparisons with one control group, more than two groups) was used for statistical analysis, which was performed in GraphPad Prism software. Statistically relevant *p*-values are depicted in the graphics.

#### Preprocessing of Fc IgG *N*-Glycopeptide Measurements

Values obtained for *N*-glycopeptide abundance were normalized to the total integrated area per IgG subclass (IgG1, IgG2 = IgG2a/c + IgG2b, IgG3) to make the values comparable between the samples. Batch correction was performed on the log-transformed values using the ComBat method (R package “sva”) to remove possible experimental variations due to a batch effect.

Derived glycosylation traits describing relative abundance of specific types of *N*-glycopeptide structures (agalactosylated, monogalactosylated, digalactosylated, monosialylated, disialylated structures, structures with bisecting GlcNAc or α1,3-linked galactose residues) were calculated in a subclass-specific manner (Supplementary Table S2). These derived traits were defined to describe the terminal sugar residues of the *N*-glycans present on IgG Fc rather than the biosynthetic pathways (meaning that “galactosylation” trait does not include sialylated *N*-glycan structures, which also possess galactose residues on 3- and 6-arms, but those are not terminal residues), as the nature of the terminal residues is important for the IgG Fc region interaction with the receptors.

#### Heat Maps and Principal Component Analysis

To visualize the differences between IgG Fc *N*-glycan trait levels in knock-out and wild-type mice, heat maps were constructed in the R programming language version 3.3.3 using the non-transformed batch-corrected data. Since the number of analyzed animals per knock-out type was small (up to 5) and the distributions of the measured values were skewed, differences of median levels of glycopeptides were used. To be able to compare glycosylation traits with different abundances, the calculated differences were normalized to the median level of a corresponding trait in the wild type. Differences were calculated separately for males and females of each background. Homogeneity of glycosylation trait variances across sample groups was assessed with Levene’s test in R (function “leveneTest” from package “car”). Additionally, to test if derived trait levels statistically differ between wild-type animals and knock-outs of corresponding background and sex, for each pair wild type–knock-out, we performed Kruskal–Wallis test followed by *post hoc* Dunn test for multiple comparisons with one control group (functions “kruskal.test” from the “stats” package and “dunn.test.control” from “PMCMR” package in R). To compare individual glycan traits between mice of BALB/c and C57BL/6 backgrounds, we performed Mann–Whitney tests in R. For all statistical tests, the false discovery rate was set at 0.05 and controlled with the Benjamini–Hochberg procedure. The adjusted *p*-values were reported along with non-adjusted. PCA was performed in R (function “prcomp” from the “stats” package). For PCA, the batch-corrected glycopeptide measurements were log-transformed due to the skewness of the distributions of their abundances.

## Results

### IgG Subclasses Concentrations

With respect to the abundance of IgG subclasses, we detected some differences between the two wild-type strains for both sexes (Supplementary Figure S1). Further along these lines, the abundance of some IgG subclasses was different between certain wild-type and knock-out mice, but the effect was most pronounced in the case of IgG3 from male FcRγ^–/–^FcγRIIB^–/–^ mice on the BALB/c background, where the difference was approximately two to fourfold, while the distributions of measurements in the two groups were not overlapping. The concentration of IgG3 appears to be much higher in the knock-out animals (Supplementary Figure S2).

### Fc-Linked IgG *N*-Glycosylation

All of the *N*-glycopeptides for which the measurements passed the quality control carried biantennary *N*-glycans with core fucose (Supplementary Table S2). Glycopeptides with bisecting GlcNAc were quantified only for IgG1. The asialylated glycoforms with terminating α1,3-linked galactose residues were observed with intensities below reliable quantification levels for all IgG subclasses and thus were not quantified. The abundance of the derived glycan traits in the wild-type and knock-out mice of BALB/c and C57BL/6 backgrounds and both sexes is shown in Supplementary Figure S3.

**FIGURE 2:**
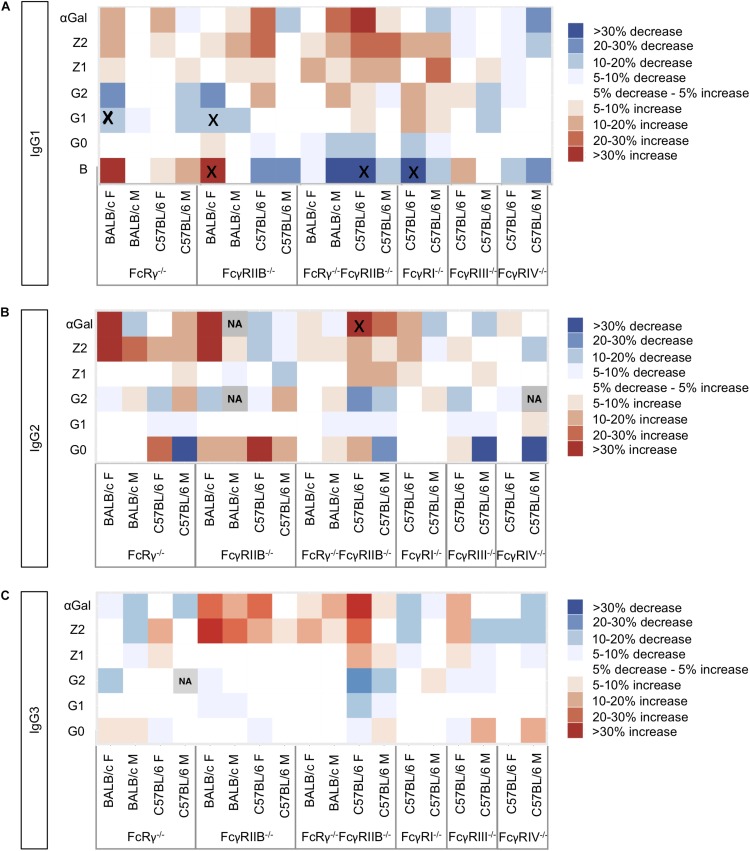
Heat maps showing relative differences between the median abundances of **(A)** immunoglobulin G (IgG)1, **(B)** IgG2, and **(C)** IgG3 fragment crystallizable (Fc) glycosylation traits in the Fcγ receptor knock-out mice and wild-type mice of the same sex and background (controls). The differences are normalized to the median value of the traits in the corresponding control group (*N* = 5–7 animals of same sex, strain, and knock-out status). Gray squares marked “NA” refer to the cases where not enough data were available to calculate median values of the trait or in cases when the variance of the trait in one group of samples was statistically different from the variance observed in the other groups (Supplementary Table S4). Black “x” denotes pairs “control–knock-out” for which the differences in a certain glycosylation trait were found to be significant in a *post hoc* test after correction for multiple tests at 0.05 level (Supplementary Table S3). Differences are normalized to the median value of the corresponding trait in the wild type.

To visualize the trends in *N*-glycosylation differences between wild-type mice and strains deficient for FcγRs, we created heat maps for each of the IgG subclasses (Figure 2) and performed Kruskal–Wallis one-way analysis of variance with *post hoc* tests (Supplementary Tables S3A,B). We were unable to compare glycosylation traits in some pairs because the variances in these traits for wild-type and knock-out groups were statistically different in Levene’e test (Supplementary Table S4). Only six derived traits were found to be statistically different between wild-type animals and some of the knock-out groups (Figure 2 and Supplementary Table S3B): monogalactosylation (G1) of IgG1 in FcRγ^–/–^ BALB/c females; bisection (B) and monogalactosylation of IgG1 in FcγRIIB^–/–^ BALB/c females; bisection of IgG1 and α1,3-galactosylation (αGal) of IgG2 in female C57BL/6 FcRγ^–/–^FcγRIIB^–/–^ mice; and bisection of IgG1 in female FcγRI^–/–^ C57BL/6 animals. For four out of these six statistically significant changes, a similar trend was observed for the opposite sex of the same background and knock-out type, although not reaching statistical significance at α = 0.05. In general, no statistically significant knock-out-related changes in *N*-glycosylation traits replicated across the two strains and two sexes (Figure 2).

**FIGURE 3:**
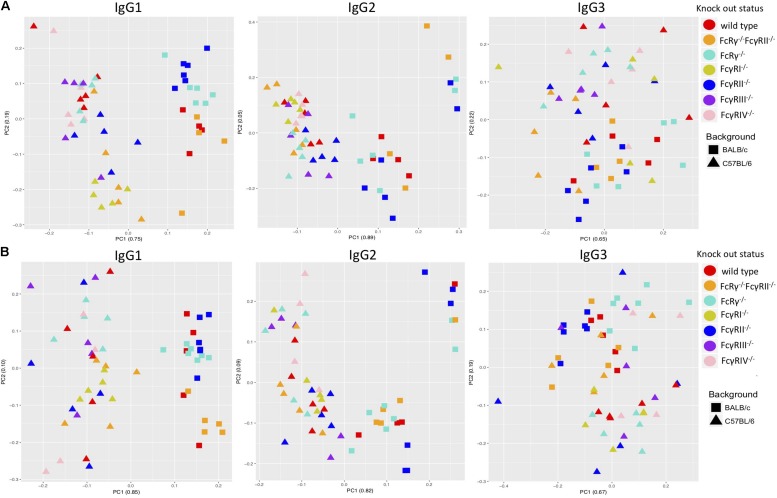
Principal component analysis of immunoglobulin G (IgG)-derived glycosylation traits in wild-type and fragment crystallizable (Fc)γ receptor (FcγR)-deficient mice, C57BL/6 versus BALB/c for females **(A)** and males **(B)**. On *x*- and *y*-axes are plotted principal components 1 and 2, respectively, with proportion of variance explained by each component shown in the brackets. *N* = 5–7 animals of same sex, strain, and knock-out status.

A PCA revealed some clustering according to knock-out type within males and females of the same strain when the two sexes were regarded separately, the most clearly observed for IgG1 glycans (Figures 3A,B). However, mice of the same knock-out type showed weaker tendency to cluster together when we compared males and females of the same strain (Supplementary Figures S4A,B).

### Strain Specificity of Fc-Linked IgG *N*-Glycosylation

**FIGURE 4:**
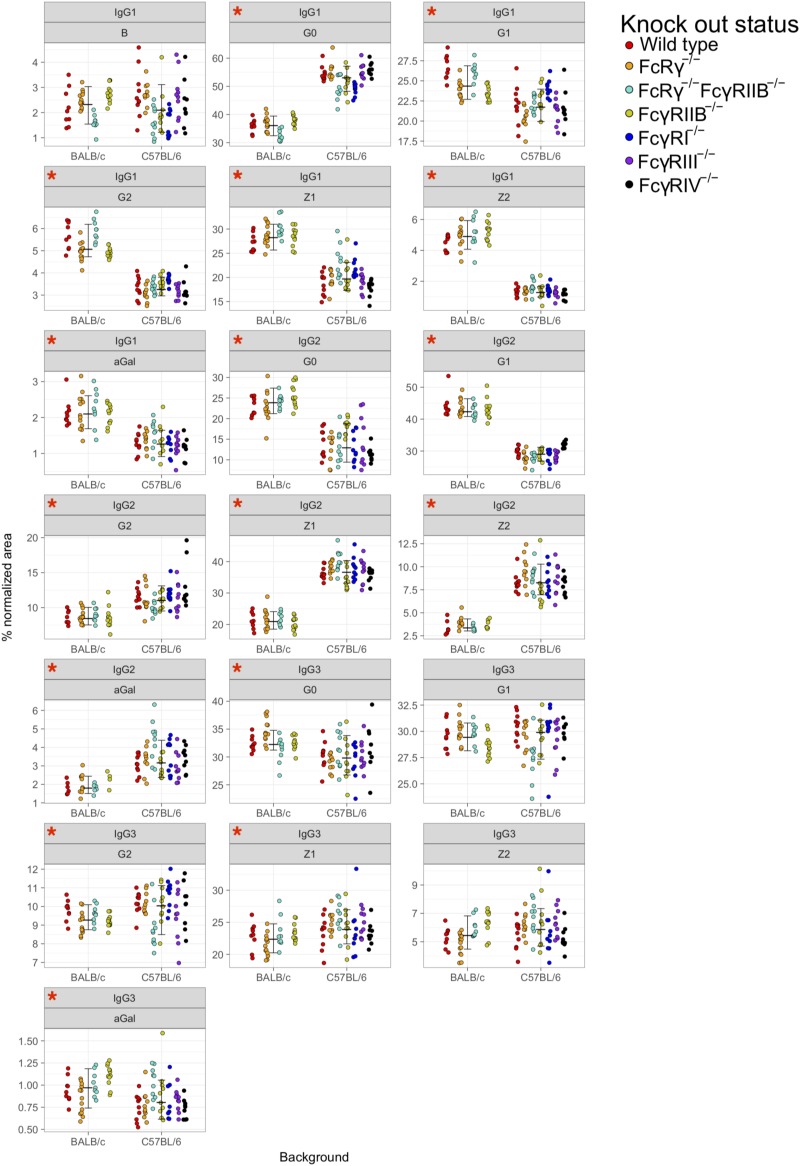
Comparison of fragment crystallizable (Fc)-linked immunoglobulin G (IgG) *N*-glycosylation in C57BL/6 and BALB/c mice. Dots represent individual data points; colors represent knock-out status of the animals. Lines with whiskers extend to the lowest data point within 1.5 *IQR of first quartile, and the highest data point within 1.5 *IQR of third quartile. Red asterisks mark the traits for which differences between the strains were statistically significant in Mann–Whitney test after Benjamini–Hochberg adjustment for multiple testing at α = 0.05. Derived traits represent relative abundance of specific types of *N*-glycan structures in the total Fc-linked *N*-glycome: structures with bisecting *N*-acetylglucosamine, B; agalactosylated structures, G0; monogalactosylated structures, G1; digalactosylated structures, G2; sialylated structures, Z; monosialylated structures, Z1; disialylated structures, Z2; structures with 1,3α-linked galactose residues. *N* = 5–7 animals of same sex, strain, and knock-out status.

Principal component analysis showed clustering according to the mouse strain background, especially for IgG1 and IgG2 *N*-glycans, less so for IgG3 (Figures 3A,B). Observed IgG glycoprofiles were indeed specific for the strain for all IgG subclasses. Mice of C57BL/6 background had a higher level of G0, lower levels of G1, G2, Z1, Z2, and αGal on IgG1; lower G0, G1, higher G2, Z1, Z2, and αGal on IgG2 (Figure 4 and Supplementary Table S5), regardless of their sex or knock-out status. Differences for some IgG3 glycosylation traits were also statistically significant, but the effect was smaller (Figure 4 and Supplementary Table S5).

## Discussion

In order to check for the existence of a possible feedback regulation of IgG *N*-glycosylation and the available repertoire of FcγRs, we examined the subclass-specific Fc IgG glycoprofiles of healthy male and female FcγR knock-out mice on C57BL/6 and BALB/c backgrounds. We observed slight differences in IgG Fc *N*-glycan profiles in different knock-outs, mostly for IgG1 *N*-glycans. It seems that the strain background and sex have a stronger effect on *N*-glycosylation of IgG Fc regions than the FcγR repertoire. Interestingly, we only observed the knock-out-related changes in IgG1 and IgG2 and none in IgG3, the only subclass that does not interact with FcγRs.

Since sex-specific IgG *N*-glycosylation was previously reported in humans (Pučić-Baković et al., 2013; Krištić et al., 2014) and mice (Krištić et al., 2018), we compared males and females separately in our analysis. In mice, females have lower levels of bisection and sialylation and higher levels of galactosylation than males (Krištić et al., 2018). Despite the aforementioned study analyzing total IgG *N*-glycome rather than subclass-specific Fc-linked *N*-glycome, we observed similar trends in wild-type BALB/c and C57BL/6 mice, although the magnitude of these differences in most cases was lower than the differences between the glycoprofiles of the two strains (Supplementary Figures S3A–C). Currently, it is believed that both galactosylation and sialylation of IgG are influenced by sex hormones, namely, estrogens, which might at least in part explain sex-specific differences (Ercan et al., 2017; Engdahl et al., 2018).

Furthermore, our results confirm that Fc-linked *N*-glycoprofiles of BALB/c and C57BL/6 mice differ in abundance of almost all major *N*-glycan types for all IgG subclasses. C57BL/6 mice tend to have a higher ratio of agalactosylated IgG1 glycoforms compared to BALB/c, while in the IgG2 Fc *N*-glycome of C57BL/6 mice, sialylated and digalactosylated structures are more abundant than in BALB/c animals. In our recent work, we found an association between the IgG *N*-glycosylation pattern and variants in the genes coding for the constant region of the IgG heavy chain (Krištić et al., 2018). BALB/c and C57BL/6 mice are well-known to have polymorphic positions in IgG (Keane et al., 2011; Yalcin et al., 2011), which we proposed as candidates for IgG *N*-glycan regulation (Krištić et al., 2018). This might explain the different IgG *N*-glycosylation profiles of the two strains reported here, which were also observed in previous LC–MS studies of murine IgG *N*-glycosylation (de Haan et al., 2017; Zaytseva et al., 2018). Moreover, it is well-known that immune responses in BALB/c mice are more dominated by Th2 cytokines, whereas immune responses in C57BL/6 mice are more skewed toward a pro-inflammatory Th1 pattern. Further studies, including transcriptomic and proteomic characterization of antibody-secreting cells, are needed to understand the regulation of IgG *N*-glycome in mouse strains, ideally on a single cell level.

Of note, there seems to be no common denominator for a possible change of IgG Fc glycosylation profile for any of the knock-outs across the two strains and two sexes: in some cases, animals of different sexes of the same strain seem to respond differently to FcγR deficiency.

In order to get a clearer picture on a possible co- or inter-dependency of IgG Fc glycosylation and the FcγR status, our results might need to be evaluated together with the differences in IgG subclass abundance. This is because the change of the IgG subclass serum level might also be a feedback corrective mechanism for FcγR deficiency.

The most prominent change of subclass abundance was observed in all three types of knock-out (FcRγ^–/–^, FcγRIIB^–/–^, and FcRγ^–/–^FcγRIIB^–/–^) for male BALB/c animals. Compared to the BALB/c male wild-type mice, knock-out animals exhibited increased levels of IgG3 (Supplementary Figure S2). However, we observed no significant differences in IgG3 Fc-linked *N*-glycoprofiles between male BALB/c wild-type mice and any of the knock-outs (Figure 2 and Supplementary Tables S3A,B).

It is important to note that most Fc *N*-glycan traits that significantly differed in FcγR-deficient animals belong to low abundant species, for example, bisected *N*-glycans on IgG1 (1–4% total normalized area) and α1,3-galactosylated *N*-glycans on IgG2 (0.5–1.5% total normalized area), and the measurement error for these species is higher than for more abundant glycopeptides (Zaytseva et al., 2018). Finally, the cohort of mice used in this experiment was healthy and immunologically not stimulated. Since IgG glycosylation in mice is known to change following immune activation (Oefner et al., 2012; Hess et al., 2013; Kao et al., 2017), the results might have been different if the study had been performed on immune-stimulated and challenged mice. Studying an adaptive immune response in individual FcγR knock-out mice *in vivo*, however, creates a high complex scenario. Thus, individual FcγRs are known to be involved in virtually all steps of an adaptive immune response, including antigen processing and presentation, affinity maturation, and the regulation of the magnitude and quality of the B cell response (Nimmerjahn and Ravetch, 2010). Therefore, many factors may indirectly impact the abundance of select IgG antibodies and IgG glycosylation variants, making it quite difficult to interpret the data. Ideally, mice with cell type-specific deletions of FcγRs may need to be studied to obtain clear insights if and how individual FcγRs impact IgG glycosylation during the establishment and maintenance phase of a humoral immune response and which cell types are involved in imprinting a certain IgG glycosylation pattern in IgG-producing plasma cells and plasmablasts. Generating informative *in vivo* model systems to answer this critical question is one of our major current efforts.

## Data Availability Statement

The datasets generated for this study are available on request to the corresponding author.

## Ethics Statement

The animal study was reviewed and approved by the District Government of Lower Franconia.

## Author Contributions

OZ, JK, and MS conducted the experiments. OZ and MP analyzed the results. OZ, MS, and MP wrote the manuscript. FN, GL, and MP conceived and supervised the experiments. All authors reviewed the manuscript and contributed in their areas of expertise.

## Conflict of Interest

GL declares that he is a founder and owner of Genos Ltd. that specializes in high-throughput glycomics analysis and has several patents in the field. OZ, JK, and MP are employees of Genos Ltd. The remaining authors declare that the research was conducted in the absence of any commercial or financial relationships that could be construed as a potential conflict of interest.

## Abbreviations

ACN: acetonitrile
C_H_2: constant heavy 2 (domain)
F: phenylalanine
Fab: fragment antigen binding
FcγR: Fcγ receptor
Fc: fragment crystallizable
GlcNAc: *N*-acetylglucosamine
I: isoleucine
IgG: immunoglobulin G
IQR: interquartile range
LC–ESI–MS: liquid chromatography–electrospray ionization–mass spectrometry
PBS: phosphate buffer saline
PCA: principal component analysis
RP SPE: reversed-phase solid-phase extraction
S/N: signal-to-noise
TFA: trifluoroacetic acid
UPLC: ultraperformance liquid chromatography
