# Supplementary material for: Fc-Linked IgG N-Glycosylation in FcγR Knock-Out Mice
Source: Front Cell Dev Biol. 2020 Mar 3;8:67. doi: 10.3389/fcell.2020.00067 (PMC7063467; doi:10.3389/fcell.2020.00067)
Supplement: Supplementary file 1 [file Data_Sheet_1.PDF]

Supplementary Figure S1

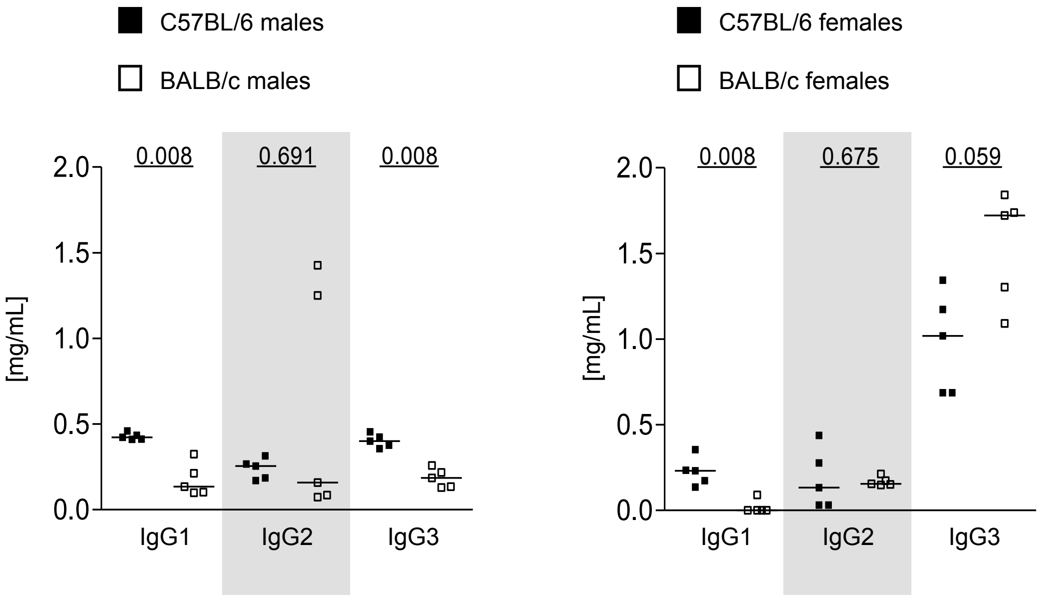

Supplementary Figure 1. The abundances of IgG subclasses in wild type male and female mice. p values obtained through Mann-Whitney test are shown.

Supplementary Figure S2

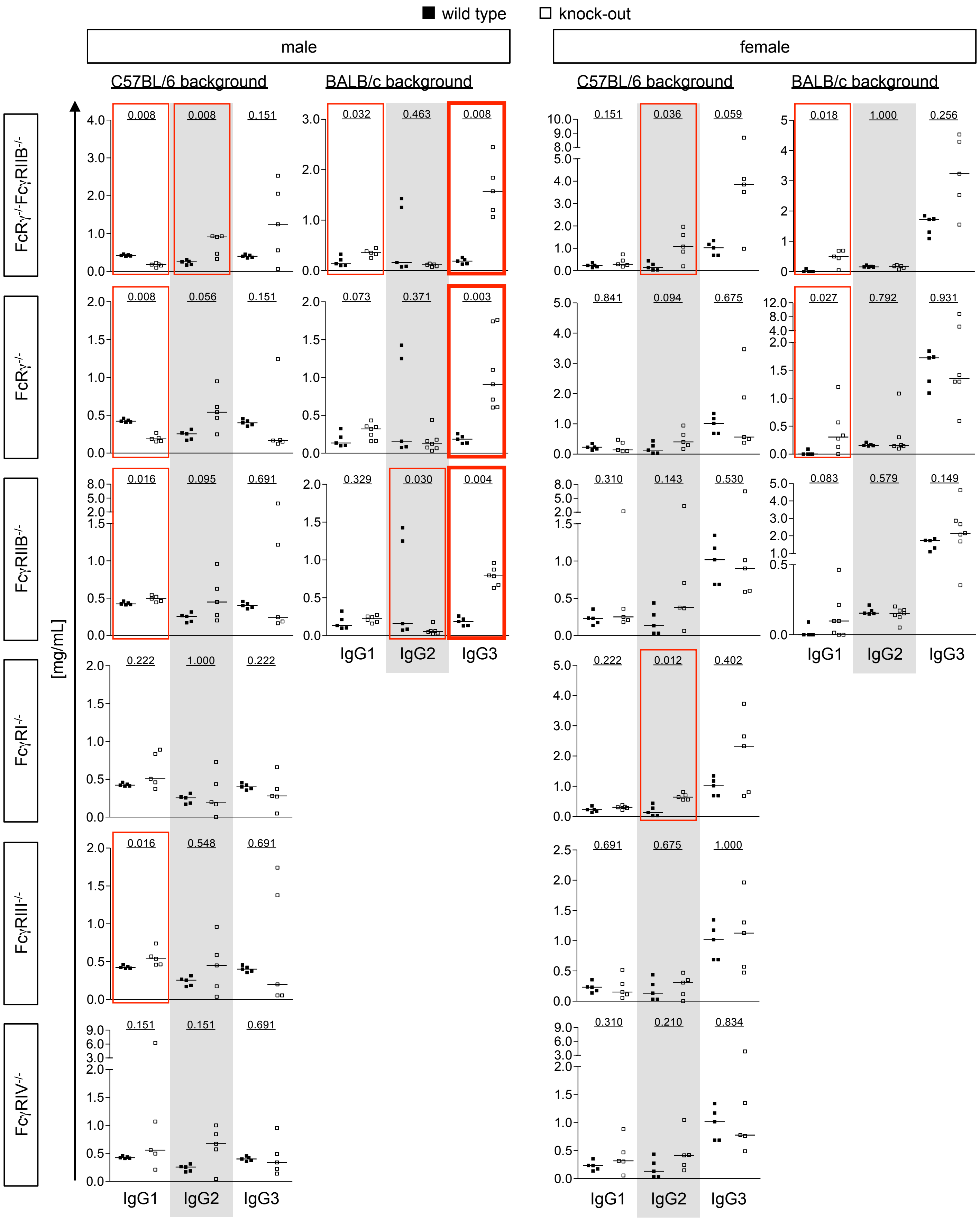

Supplementary Figure 2. The abundances of IgG subclasses in wild type vs. knock-out mice. p values obtained through Mann-Whitney test are shown. Groups for which the differences were statistically significant at 0.05 are marked by red frames.

Supplementary Figure S3A.

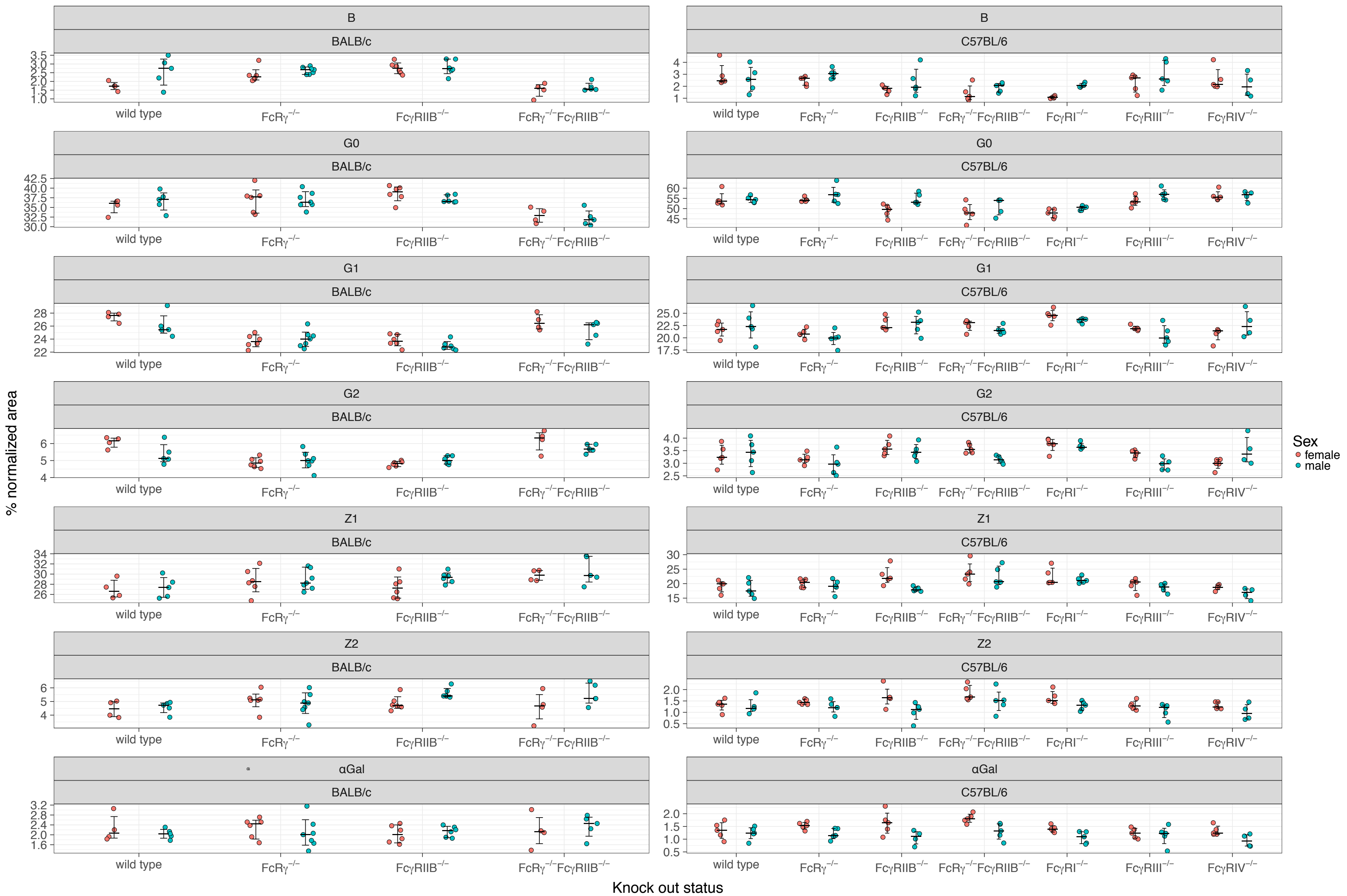

Supplementary Figure 3A. Distribution of Fc-linked IgG1 glycosylation traits in male and female C57BL/6 and BALB/c mice of wild type and deficient for certain types of FcγRs.

Supplementary Figure S3B.

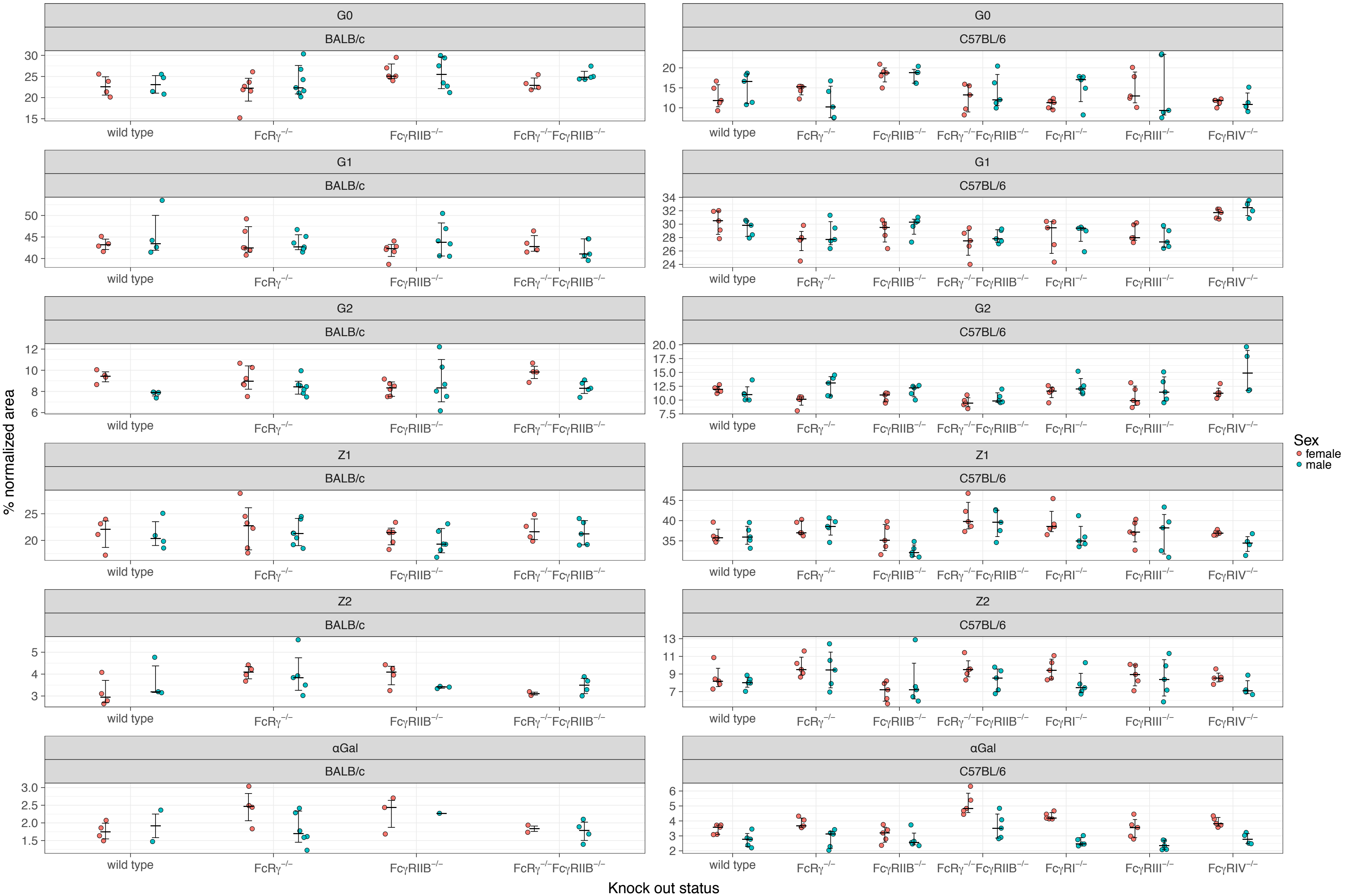

Supplementary Figure S3B. Distribution of Fc-linked IgG2 glycosylation traits in male and female C57BL/6 and BALB/c mice of wild type and deficient for certain types of FcγRs.

Supplementary Figure S3C.

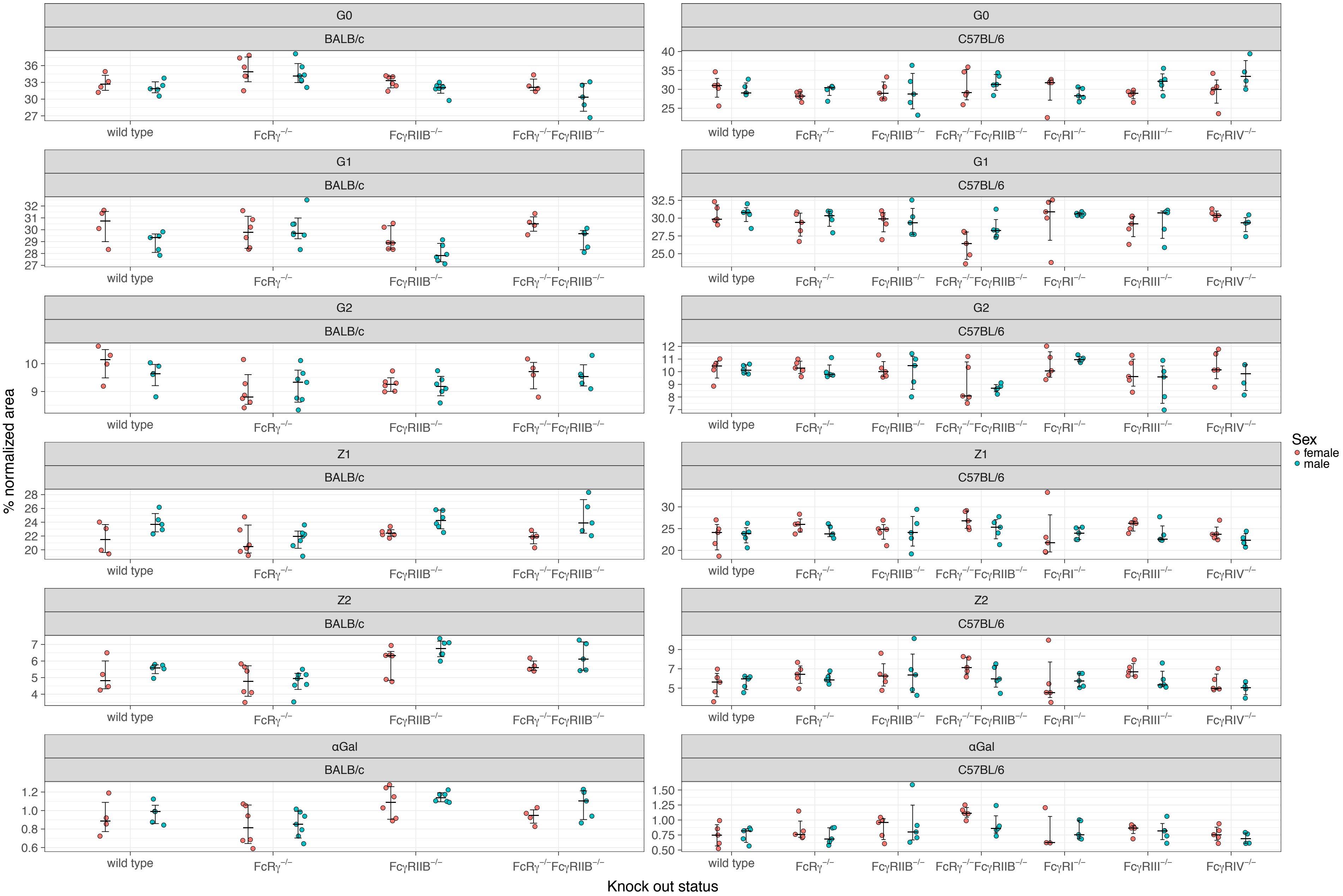

Supplementary Figure S3C. Distribution of Fc-linked IgG3 glycosylation traits in male and female C57BL/6 and BALB/c mice of wild type and deficient for certain types of FcγRs.

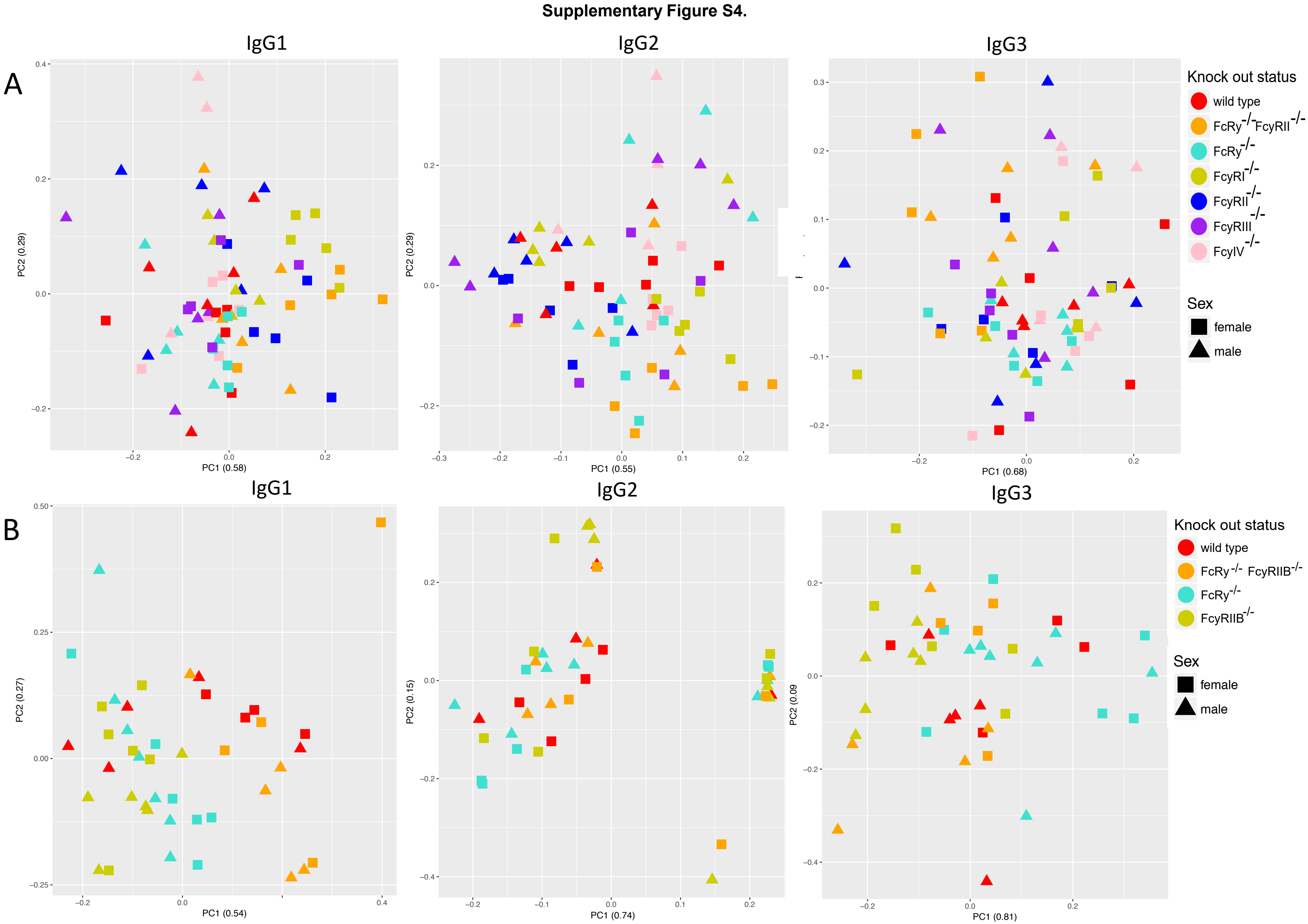

Supplementary Figure S4. Principle component analysis of IgG derived glycosylation traits in wild type and FcγR deficient mice, females vs males for C57BL/6 (A) and BALB/c (B). On x- and y-axes are plotted principal components 1 and 2 respectively with proportion of variance explained by each component in the brackets.
