# Supplementary material for: Fc-Linked IgG N-Glycosylation in FcγR Knock-Out Mice
Source: Front Cell Dev Biol. 2020 Mar 3;8:67. doi: 10.3389/fcell.2020.00067 (PMC7063467; doi:10.3389/fcell.2020.00067)
Supplement: Supplementary file 3 [file Table_2.DOCX]

**Supplementary Table S2. N-glycan structures detected on Fc regions of murine IgG and derived glycosylation traits.**


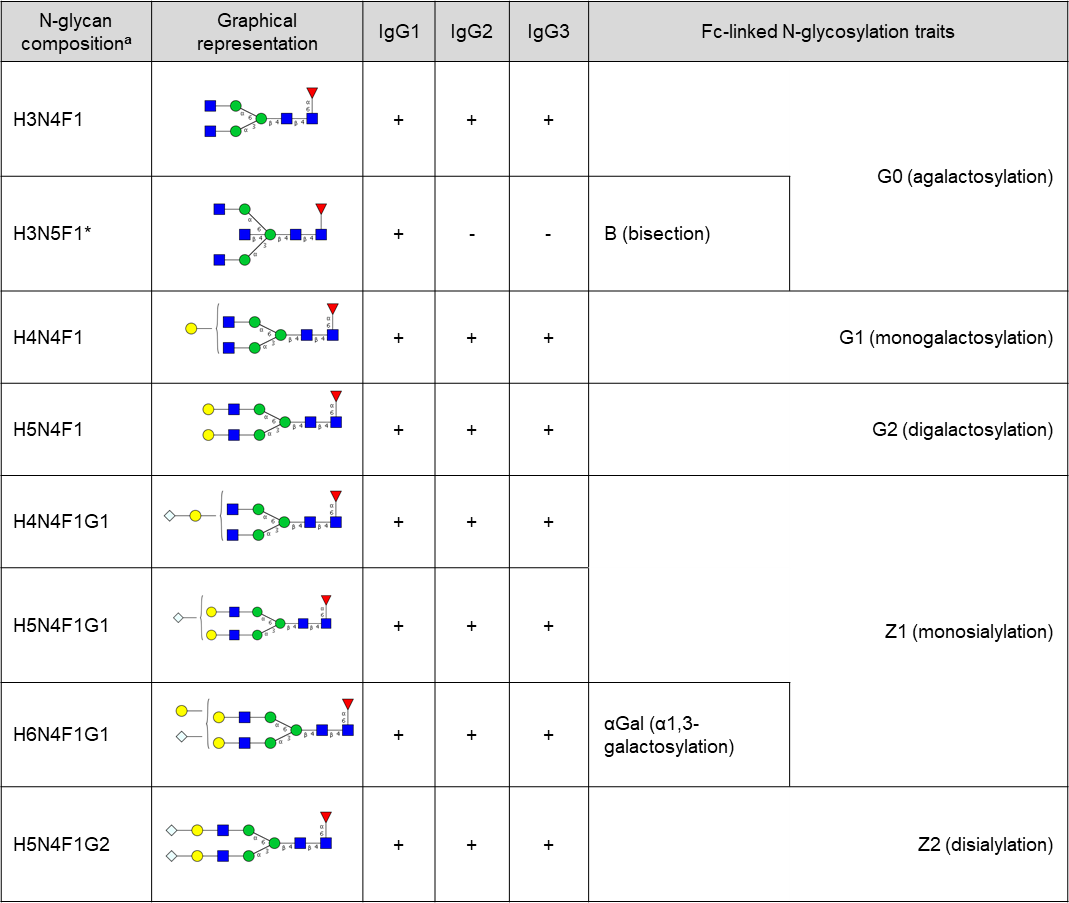


*N*-Glycolylneuraminic acid

Galactose

*N*-Acetylglucosamine

Mannose

Core fucose
